# Siglec Signaling in the Tumor Microenvironment

**DOI:** 10.3389/fimmu.2021.790317

**Published:** 2021-12-13

**Authors:** Eline J. H. van Houtum, Christian Büll, Lenneke A. M. Cornelissen, Gosse J. Adema

**Affiliations:** ^1^ Radiotherapy & OncoImmunology Laboratory, Department of Radiation Oncology, Radboud Institute for Molecular Life Sciences, Radboud University Medical Center, Nijmegen, Netherlands; ^2^ Hubrecht Institute, Royal Netherlands Academy of Arts and Sciences (KNAW), Utrecht, Netherlands; ^3^ Copenhagen Center for Glycomics, Departments of Cellular and Molecular Medicine, Faculty of Health Sciences, University of Copenhagen, Copenhagen, Denmark

**Keywords:** Siglecs, sialic acids, sialoglycans, tumor microenvironment, immune checkpoint, cancer, immunotherapy

## Abstract

Sialic acid-binding immunoglobulin-like lectins (Siglecs) are a family of receptors that recognize sialoglycans – sialic acid containing glycans that are abundantly present on cell membranes. Siglecs are expressed on most immune cells and can modulate their activity and function. The majority of Siglecs contains immune inhibitory motifs comparable to the immune checkpoint receptor PD-1. In the tumor microenvironment (TME), signaling through the Siglec-sialoglycan axis appears to be enhanced through multiple mechanisms favoring tumor immune evasion similar to the PD-1/PD-L1 signaling pathway. Siglec expression on tumor-infiltrating immune cells appears increased in the immune suppressive microenvironment. At the same time, enhanced Siglec ligand expression has been reported for several tumor types as a result of aberrant glycosylation, glycan modifications, and the increased expression of sialoglycans on proteins and lipids. Siglec signaling has been identified as important regulator of anti-tumor immunity in the TME, but the key factors contributing to Siglec activation by tumor-associated sialoglycans are diverse and poorly defined. Among others, Siglec activation and signaling are co-determined by their expression levels, cell surface distribution, and their binding preferences for *cis-* and *trans*-ligands in the TME. Siglec binding preference are co-determined by the nature of the proteins/lipids to which the sialoglycans are attached and the multivalency of the interaction. Here, we review the current understanding and emerging conditions and factors involved in Siglec signaling in the TME and identify current knowledge gaps that exist in the field.

## Introduction

Humans express 14 members of the Sialic acid-binding immunoglobulin-like lectins (Siglecs) that are divided in two subfamilies based on their sequence similarity and evolutionary conservation. Siglec-1 (also known as sialoadhesin and CD169), Siglec-2 (also known as CD22) Siglec-4 and Siglec-15 have clear orthologous in mammalian species (1–4). The CD33-related Siglecs (Siglec-3 also known as CD33, Siglec-5 to -11, -14 and -16) evolved more rapidly among species. These transmembrane receptors are mainly present on immune cells, but also on other cell types such as trophoblasts, myelin-forming cells, and stromal cells (1, 3, 4). Siglecs bind their ligands *via* an extracellular N-terminal V-set domain. Intracellularly, most Siglecs (Siglec-3, -5 to -9, and -11) harbor a combination of a membrane proximal ITIM (immunoreceptor tyrosine-based inhibition motif) domain and a membrane distal ITIM-like domain (1–3). Upon Siglec activation, this ITIM domain is phosphorylated by Src family kinases. Phosphorylation subsequently leads to recruitment of SH2-domain containing phosphatases SHP-1 and/or SHP-2, which dephosphorylate downstream components of immune stimulatory pathways, thus inhibiting cellular activation. Interestingly, three Siglecs (Siglec-14, -15 and -16) do not contain ITIM domains, but have a positively charged residue in their transmembrane domain that enables them to complex with ITAM (immunoreceptor tyrosine-based activation motif) containing adaptor proteins, such as DAP10 or DAP12 (2, 3, 5). This leads to recruitment of protein kinases that can phosphorylate downstream targets, eventually triggering downstream signaling pathways (1–3, 5). Besides ITIM- and ITAM-containing Siglecs, Siglec-1 does not contain known intracellular signaling motifs.

**Figure 1:**
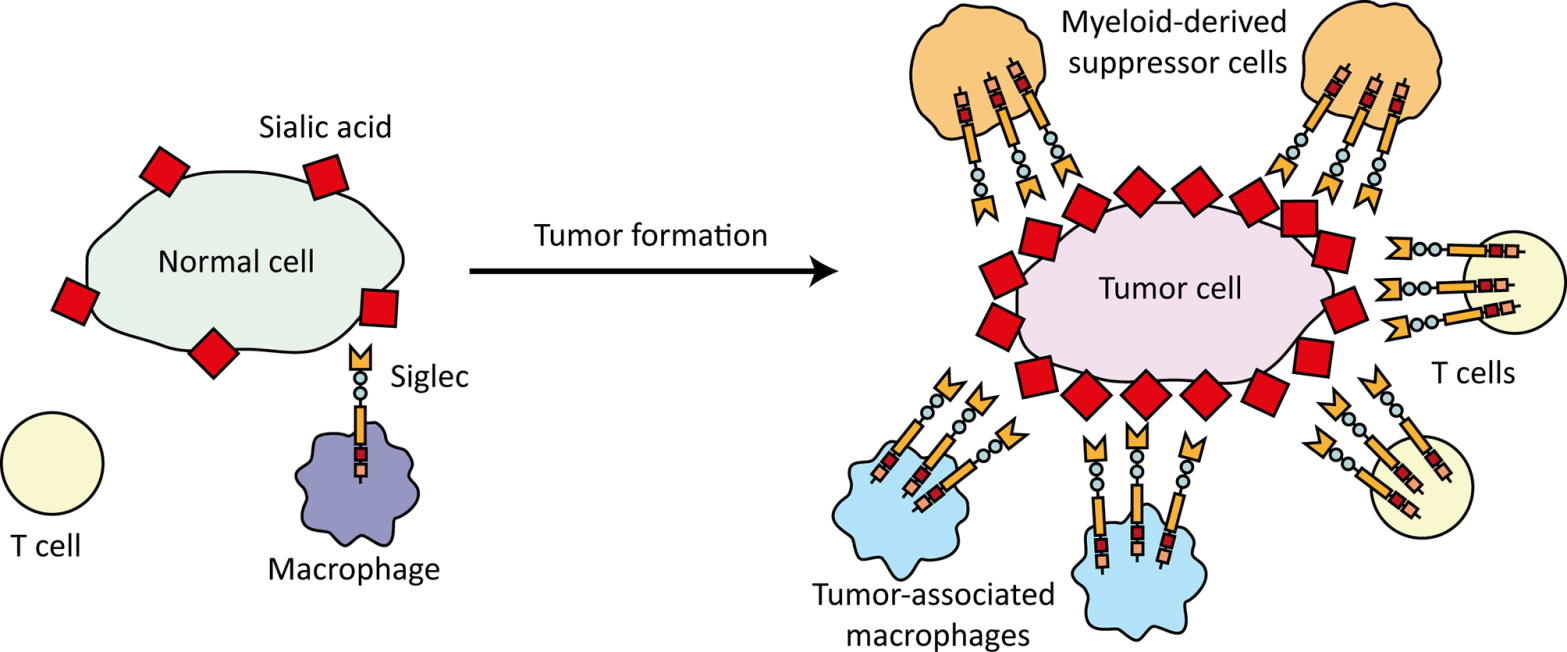
Schematic overview of changes in the Siglec-sialoglycan axis upon tumor formation. Immune cells circulating in healthy tissue express Siglecs and cells in this environment express ‘normal’ sialic acid (red diamonds) types and levels. Upon tumor development, immune cells infiltrate the TME, such as tumor-associated macrophages, T cells, and myeloid-derived suppressor cells (and many other types). These immune cells display changed and partially enhanced Siglec expression levels and can interact with tumor-associated sialoglycans that are formed and often increased as result of altered glycosylation in cancer cells. Siglec-sialoglycan interactions in the TME can lead to functional modulation of infiltrating immune cells and the anti-tumor immune response.

The ligands for the Siglecs are constituted by glycans, highly diverse biomolecules composed of various monosaccharides that are linked to membrane bound and secreted glycoproteins, glycolipids, and as recently suggested also small noncoding glycoRNAs (3, 4, 6–9). Siglecs selectively recognize glycans that contain one or more negatively charged sialic acid residues, so called sialoglycans (1, 6). Sialoglycans are present on virtually every human cell, but are aberrantly expressed on tumor cells where they contribute to tumor growth and progression, including metastasis (10). Interestingly, Siglec expression has been reported to be enhanced and/or induced on other cells within the TME, including immune cells (11–16). Concomitantly, the combination of altered Siglec expression on immune cells and aberrant sialoglycan expression on tumor cells, could possibly lead to strong Siglec activation and resulting in immune cell inhibition within the TME (**Figure 1**). The enhanced sialoglycans expression within the TME is clearly described in literature, however, the evidence that this directly translates into more Siglec ligand expression and Siglec signaling remains scarce.

The structure and signaling motifs of Siglec receptors show great resemblance to the well-known inhibitory receptor PD-1 (3, 17) that is used as target in immunotherapy of cancer. Moreover, Siglecs are often co-expressed on T cells with other inhibitory receptors, such as PD-1, TIM-3 and LAG-3 (11). Therefore, the immune inhibitory Siglecs are currently envisioned as potential immune checkpoint receptors that can be targeted in cancer (17–20). Despite Siglecs emerging as an attractive target for cancer immunotherapy, the exact cell and glycobiological conditions that trigger, regulate and control Siglec signaling in the TME remain largely elusive. Here, we will review the current knowledge on factors (co-)determining Siglec signaling and discuss knowledge gaps regarding this Siglec-sialoglycan signaling axis focusing on the TME.

## Siglec Expression Within the TME

Siglec family members are expressed by most cell subsets of the human immune system and by several tissues and cell types outside of the immune system (1–4). Some Siglecs have immune cell type specific expression and can therefore be used as differentiation marker such as Siglec-1 that is found mainly on macrophages, Siglec-2 that is specifically expressed by B cells, Siglec-3 that marks cells from the myeloid lineage including microglia cells, or Siglec-8 that is found predominantly on eosinophils (3, 8). In the TME, focus has been mainly on the inhibitory Siglecs-7, -9, and -10 as well as the activating Siglec-15. Siglec-7 and -9 are abundantly present on natural killer (NK) cells with Siglec-7 being a pan-marker for human NK cells and Siglec-9 being present on a population of CD56^dim^ CD16^+^ NK cells (21). Strong interactions of these Siglecs with synthetic and natural multivalent sialoglycan ligands appears to be sufficient to inhibit NK cell activation (22). Accordingly, blocking Siglec-7 and -9 interactions with sialoglycans using monoclonal antibodies increased killing of several tumor cell lines (K562 and HeLa) by peripheral blood NK cells (21). Another example of a Siglec-mediated immune modulatory effect was observed in T cells. Only a small percentage (1-3%) of peripheral blood CD8^+^ T cells express Siglec-7 and/or Siglec-9 (11, 23). Remarkably, Siglec-9 expression on CD8+ T cells present in peripheral blood and tumor tissue of non-small cell lung cancer (NSCLC) patients is upregulated up to 25% and 40% respectively (11). Similarly, tumor-infiltrating CD8^+^ T cells in melanoma patients have also been found to display enhanced levels of Siglec-9 (13). Functionally, Fab fragments targeting Siglec-9 could modulate activation and IL-2 production of staphylococcal enterotoxin B stimulated tumor-infiltrating lymphocytes of primary NSCLC patients (11). Siglec-9 antibodies could increase cytotoxicity of healthy donor CD8^+^ T cells against anti-CD3-loaded P815 tumor cells and enhance IFNγ and TNFα production (13). Besides NK and T cells, immune modulation of Siglec-9 expressing myeloid cells has also been observed. For instance, Rodriguez et al. showed that pancreatic ductal adenocarcinoma-derived sialic acid can cause monocytes to differentiate to macrophages, which was prevented using anti-Siglec-7 and anti-Siglec-9 blocking antibodies (24).

Siglec-15 has emerged as an interesting target for cancer immunotherapy (25). Siglec-15 contains a positively charged residue in its transmembrane domain and is therefore regarded as an activating Siglec (26). Siglec-15 expression was observed on tumor-associated macrophages (TAMs) in the TME of human lung and rectal adenocarcinoma and human hepatocellular carcinoma (27). The interaction between Siglec-15 expressing monocytic THP-1 cells and sialyl-Tn expressing H157 human lung carcinoma cells enhanced TGF-β production. Next to its expression on myeloid cells, Siglec-15 was observed on stromal cells and even on tumor cells (28). Siglec-15 expression on tumor cells was verified by others on several types of cancer, such as lymphoma, gastric cancer and acute myeloid leukemia (29–31). Similarly, Siglec-8 has been reported to be expressed by breast cancer cells (32). Next to that, several Siglecs were found to be expressed on hematological cancers (33–35). Siglec-6 expression was observed on acute myeloid leukemia cell lines, primary acute myeloid leukemia blasts, transformed B-cells in chronic lymphocytic leukemia (33). Siglec-2 expression on acute lymphoid leukemia has also been documented and Siglec-3 is found on blasts in nearly all acute myeloid leukemia patients (34, 35). Besides Siglec expression on immune cells and on tumor cells, Siglecs might also be expressed within the stromal compartment, as Siglec-11 expression was found on human and chimpanzee ovarian stromal cells (36). However, the influence of the stromal compartment on the Siglec-sialoglycan signaling axis remains largely unexplored.

### Regulation of Siglec Expression

How Siglec expression is being triggered and regulated is still largely unknown, including the factors contributing to the enhanced expression in the TME (12). Siglec-10 was found to be upregulated on CD52^+^ CD4^+^ T cells upon CD3/CD28 activation (37, 38). Therefore, one factor contributing to enhanced Siglec expression is cellular activation, but this was not investigated within the TME context. Another way tumor cells influence Siglec-10 expression on immune cells has been studied by Li et al. (39). They showed that extracellular vesicles (EVs) isolated from ascites from epithelial ovarian cancer patients could induce Siglec-10 expression on Jurkat T cells. Functionally, treatment of Jurkat T cells with tumor cell derived EVs inhibited PMA/ionomycin-induced protein kinase C activity and impaired phosphorylation of tyrosine kinase zeta-chain-associated protein kinase 70 after activation with anti-CD3, but direct evidence for Siglec signaling is lacking.

Siglec-15 expression was found to be downregulated by IFN-γ on monocyte-derived macrophages and RAW264.7 cells and to be dependent on macrophage-colony stimulating factor (28). Interestingly, also Siglec-1 expression was shown to be influenced by cytokines, as it could be induced by IFN-α and IFN-γ (40). Moreover, a few studies have observed that immune suppressive cytokines can influence Siglec expression, which might explain how the immune suppressive TME affects Siglec expression on immune cells. For instance, De Saint Jean et al. (2017) reported that TGF-β1 can enhance Siglec-1, but not Siglec-5, -7, -9, -10 and -14, expression on monocyte-derived dendritic cells (41). Nagase et al. did not observe increased Siglec-1 expression on the monocytic THP-1 cell line by treatment with TGF-β, but rather after stimulation with IL-1β and TNF-α (42). Another study showed that high-expressing Siglec-1 peripheral blood mononuclear cells (PBMC) derived macrophages (CD14^+^ monocytes differentiated with 10% human AB serum) downregulate Siglec-1 expression upon treatment with tumor culture supernatant from HepG2 cells and upon treatment with recombinant human TGF-β, but not with recombinant human TNF-α or IL-10 (43). Lastly, Calzada-Wack et al. showed that IL-10 treatment of *in vitro* cultured blood monocytes resulted in decreased Siglec-3 expression (44). Furthermore, it has been shown that Siglec-expressing cells can also be recruited to the TME, for example the TME of gliomas can contain many myeloid-derived suppressor cells (MDSCs) that express Siglecs (45). These data show that both monocytic MDSCs and polymorphonuclear MDSCs from the glioma TME express Siglec-3, -5, 7 and -9. Potentially, TME characteristics such as metabolic shift, hypoxic areas and changes in stromal compartment could all affect Siglec expression and signaling in the TME.

One component that might influence Siglec expression in general and in the TME in particular is the dynamics of their recruitment to the membrane, internalization, recycling, and degradation. Siglec-2 has been shown to be constitutively endocytosed, and several Siglec receptors (Siglec-1, Siglec-2, Siglec-3) have been demonstrated to be internalized after ligand binding or antibody crosslinking, and it was shown that Siglec-2 internalization was controlled by tyrosine residues Tyr (843) or Tyr (863) in its intracellular ITIM motif (46–52). Phosphorylation of the ITIM motifs can induce an intracellular pathway that eventually leads to Siglec degradation. Suppressor of cytokine signaling 3 and Cbl, a RING finger-containing E3 ligase, can bind the phosphorylated ITIM of Siglec-3 (51, 53). This leads to ubiquitination and proteasomal degradation of Siglec-3.

Regarding Siglec recruitment to the membrane, a recent study by Chen et al. showed that N-glycosylation of Siglec-15 affects its localization (54). Treatment with a variety of lysosome inhibitors showed that Siglec-15 was degraded in a lysosome-dependent manner in Siglec-15 overexpressing HEK293T cells. Moreover, inhibition of glycosylation using tunicamycin diminished transportation of Siglec-15 to the cell membrane and promoted lysosomal degradation of the receptor. Interestingly, this process was regulated by glucose uptake. As we will touch upon later, differences in glucose uptake are observed within the hypoxic TME, so how this affects Siglec expression is an interesting topic for future studies (55).

A few studies have observed soluble Siglec receptors, indicating that receptor shedding could contribute to Siglec expression as well. Ito et al. for instance showed presence of the ectodomain of Siglec-9 in the secretome of serum-free conditioned medium from stem cells derived from human exfoliated deciduous teeth (56). Moreover, soluble Siglec-8 has been observed in serum from patients with different forms of eosinophilia (57). However, the contribution and effect of Siglec receptor shedding in the TME remains to our knowledge largely unstudied.

In summary, recent studies support the notion that Siglec expression on immune cells in the TME is enhanced compared to normal physiology. These changes in Siglec expression may be the result of altered cellular signaling and activation, secreted factors such as EVs and cytokines in the TME, as well as altered expression, glycosylation, internalization, and degradation dynamics of Siglecs themselves. In addition, to upregulation of expression, the enhanced presence of Siglec expressing cells could also be due to preferential recruitment to the TME. More detailed investigation is required to understand how Siglec expression and function is controlled under steady state conditions and within the TME, including the transcription factors and epigenetic mechanisms involved.

## Sialoglycan Ligands for Siglecs in the TME

Both healthy cells and cancer cells generate structurally highly diverse sialoglycans that are displayed on the cell surface and on secreted glycoproteins and glycolipids. Early studies already reported changes in sialoglycan expression during cancer development and found sialoglycans on multiple distinct cell types within the TME, including tumor cells and immune cells (6, 10, 15, 16, 58–60). Sialic acids were described to play a role in tumor progression, for instance by masking antigens on tumor cells to prevent immune cell recognition, by avoiding complement activation, hindering physical interactions with immune cells, and by functioning as ligand for immunosuppressive Siglecs (10). As reviewed extensively elsewhere, there are many different ways employed by tumors to change their glycosylation patterns including sialic acid levels (15, 61–65). In addition to tumor cells, Siglec ligands have been observed in the stromal compartment of the TME. For instance, Siglec-7 and -9 ligands were identified on human bone marrow-derived mesenchymal stromal cells (66). Interestingly, Siglec-9 ligands were higher expressed on cancer-associated fibroblasts than on normal fibroblasts. In mice, Siglec-E ligands were found on mesenchymal stromal cells in the presence of tumor conditioned medium and on aortic endothelial cells after lipopolysaccharide (LPS) treatment (67). Siglec-9 ligands were also expressed on human aorta and HUV-EC-C endothelial cells at high glucose levels and Siglec-10 was shown to bind the endothelial protein VAP-1 (67, 68). However, expression of Siglec ligands on stromal cells needs further confirmation *in vivo*.

**Figure 2:**
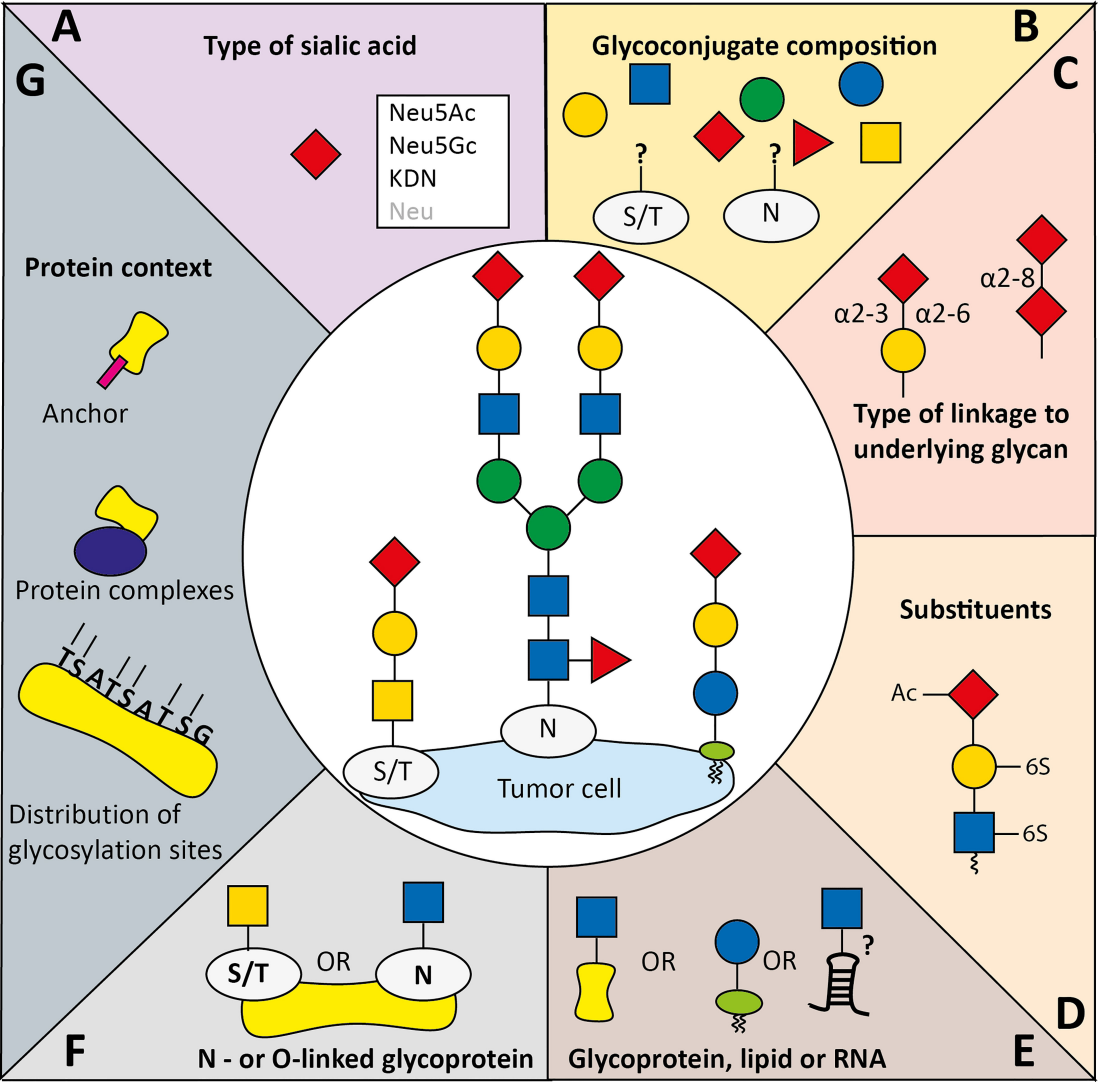
Variables implicated in co-determining Siglec specificity for sialoglycans. Siglecs display specific binding preferences for sialoglycans which is among others determined by **(A)** the type of sialic acid, **(B)** the glycan composition, and **(C)** the type of glycosidic linkage of sialic acid to the penultimate glycan. Siglec binding preferences to sialoglycans can be (co-)determined by **(D)** glycan modifications to the sialic acids or other glycans (Ac: acetylation, 6-S: 6-O-sulfation), **(E)** the glycan carrier backbone which can be lipids, proteins, and potentially RNA, **(F)** the type of protein glycosylation (O-linked to the oxygen atom in serine or threonine, or N-linked to the nitrogen atom in asparagine). Besides, **(G)** the protein backbone, including distribution of glycosylation sites, complexing with other proteins and specific (glycosylated) anchors can provide specific binding context for Siglecs.

Printed arrays of synthetic glycans and cell-based arrays have revealed that the individual Siglec family members have unique and partially overlapping binding specificities for distinct ‘healthy’ or cancer-associated sialoglycan structures (3, 8, 9, 69–71). However, the binding epitome for all the human Siglecs and ligand dynamics in the TME is of tremendous structural diversity and has not been fully mapped (**Figure 2**). Arguably, the glycosylation changes in the TME that result in specific Siglec binding or potentially abrogate binding events largely determine signaling of inhibitory and activating Siglecs.

It is important to note that the increased sialylation within the TME is mostly demonstrated with plant lectin staining and gene expression data of the enzymes involved in the sialylation pathway. Recombinant Siglec proteins are used to show whether these changes in sialoglycans also lead to more Siglec ligand binding, however, data demonstrating how this translates into more Siglec signaling is lacking or mostly indirect.

Many structural aspects of sialoglycans can contribute to Siglec binding. Here, we will summarize the current knowledge on factors implicated in Siglec preferences for specific sialoglycans and identify factors regulating expression of these sialoglycans within the TME (**Figure 2**).

### Siglec Binding Preferences for Sialic Acids

All functionally expressed human Siglecs contain a conserved arginine residue in the V-set domain that forms a salt bridge with the carboxylate group of sialic acids and is essential for sialic acid recognition (1, 4). Sequence diversity between the Siglecs further co-determines their glycan fine binding specificities. The term sialic acids refers to a large family of related sugar derivatives that share the same 9-carbon backbone which can undergo extensive natural modification (6, 72). The four core sialic acids are *N*-acetylneuraminic acid (Neu5Ac), *N*-glycolylneuraminic acid (Neu5Gc), 3-deoxy-d *-*glycero*-* d *-*galacto*-*nonulosonic acid (KDN), and neuraminic acid (Neu) which can be further altered by additional modifications (**Figures 2A**–**D**) (6). Generally human Siglecs prefer Neu5Ac, the most abundant sialic acid in human, but some including human Siglec-2 recognize both Neu5Ac and Neu5Gc with similar affinity (73–76).

Next to the sialic acid core structure, Siglecs feature specific preference for how the sialic acid is linked (α2-3, α2-6, or α2-8 linkages) to underlying glycans. For instance, Siglec-2 has clear preference for α2-6-linkages, Siglec-9 prefers α2-3-linked sialic acid, and Siglec-7 seems to have preference for both α2-3 and α2-8 linkages (3, 9, 69, 71, 77) (**Figure 2C**). Altered expression of sialyltransferase enzymes during cancer development could change the sialic acid linkage types towards one or the other. For instance, ST6GalNAc-I has been associated with cancer, which leads to α2-6 linked sialyl Tn antigen that has been shown to be recognized by Siglec-7 (9, 78, 79). Siglec-15 has also been suggested to bind Sialyl Tn, although a recent study could not confirm this interaction (9, 26, 80).

### Modification of Monosaccharides

Besides the type of the sialic acid core, Siglec binding to its ligands can be influenced by modification of monosaccharides within the sialoglycan (**Figure 2D**) (3, 70). For instance, galactose 6-*O*-sulfation has been identified as critical component for Siglec-3, -5, -8 and -14 binding and 6-O-acetylation was shown to be important for Siglec-9 binding (9, 81–83). Accordingly, expression of the sulfotransferase CHST1 in HEK293 cells that installs a 6-*O*-sulfo group to galactose induced binding of recombinant Siglec-3, -8, and -15 and largely enhanced Siglec-7 binding (9, 82). In line with this, several studies showed that Siglec-7 binds the carbohydrate sialyl 6-sulfo Lewis^x^ that is expressed by non-transformed colonic cells, rather than sialyl Lewis^x^, which is expressed by tumor cells (23, 84, 85). This could result in decreased Siglec activation and concomitant enhanced inflammation (23). It was demonstrated that Siglec-7 on macrophages exerts an immunosuppressive effect upon ligand binding. Besides sulfation, other modifications have also been described to affect Siglec binding. For instance, a study that investigated binding of recombinant Siglec Fc chimeras to biotinylated polyacrylamide probes that were conjugated to sialylated glycans found that fucosylation of sialoglycans can reduce Siglec binding (86). Furthermore, sialic acid O-acetylation has been shown to generally abrogate Siglec binding to sialoglycans (86, 87). Using CRISPR-Cas9-mediated knockouts of Sialic acid acetylesterase and Sialic acid acetyltransferase, a recent study by Grabenstein et al. showed that Siglec-7, -9, -10 and -11 binding to HCT 116 colon cancer cells and A549 lung cancer cells was decreased upon knock out of the Sialic acid acetylesterase (88). Furthermore, knock out of Sialic acid acetylesterase resulted in enhanced NK cell-mediated cytotoxicity in colon and lung cancer cells.

These studies suggest that glycan modifications such as sulfation and acetylation and the regulation of expression and activity of glycan modifying enzymes in the TME can have a strong impact on Siglec interactions with sialoglycans. Further studies into the presence of these modification in the TME compared to healthy tissue and effects on Siglec signaling could reveal a major role for glycan modifications in the tumor immune response.

### Influence of Protein/Lipid Context on Siglec Binding

Sialoglycans are attached to different backbones, such as proteins/peptides (either N-linked to asparagine or O-linked to serine or threonine) or lipids (89, 90). Recently Flynn et al. provided evidence that sialoglycans can be attached to conserved small noncoding RNAs (7) (**Figures 2E, F**). Cell surface glycoRNAs were proposed to specifically interact with Siglec-11 and -14, but not with Siglec-5 which shares the same V-set domain sequence with Siglec-14 (7, 91). Siglecs can harbor a very distinct binding preferences for N- and O-linked sialoglycans which is presumably mediated by the variable Siglec C-C’ loop (3, 4, 9, 59, 70, 71). Ligand interactions with Siglec-7, and probably the other Siglecs, induced conformational changes in the sialic acid binding site and in the C-C’ loop resulting in further interactions with the glycosphingolipid core region itself, indicating that the lipid context is of importance for Siglec-7 binding (92, 93). Furthermore, Siglec-7 binds the two linked sialic acid residues (Neu5Acα2,8Neu5Acα) and most of the other sugar moieties of a GT1b analog, demonstrating that not only the sialic acid monomer is important for binding, but the underlying glycan as well (93). More Siglec preferences have extensively been reviewed elsewhere (3, 8). Thus, Siglec binding is determined by both the context of the protein or lipid backbone and the structure of the accompanied glycans.

The hypothesis that the protein context is important for Siglec binding can be demonstrated by two specific ligands, CD24 and CD52, that were identified for Siglec-10 and which are highly similar in structure (94–96). Both are relatively small glycoproteins, consisting of 31-35 (CD24) or 12 amino acids (CD52) (97–99). They both harbor a glycosylphosphatidylinositol anchor (GPI anchor) and can be released from the cell membrane by the action of phospholipases. Interestingly, the GPI anchor, consisting of a phospholipid tail, a glycan core and a phosphoetanolamine linker to which the protein is attached, contains a glycolipid that can be sialylated as well (100–102). Therefore, CD24 and CD52 are glycoproteins attached to a glycolipid, which makes their context highly interesting for future studies. CD24 and CD52 have been shown to complex with HMGB1, adding even an additional layer of complexity, as binding of Siglec-10 to CD52 has even been shown to be enhanced by HGMB1 (95, 103, 104). Therefore, the context of the glycoprotein can cause binding by third proteins that influence Siglec binding. Both CD24 and CD52 have been shown to inhibit macrophage and T cell immune cell function through Siglec-10 binding (94, 96, 103). Studies showed that the interaction affects a diverse set of processes, such as phagocytosis by TAMs, Lck and ZAP-70 phosphorylation in CD4^+^ T cells and cytokine secretion by activated T cells.

In summary, the protein/lipid context of a Siglec ligand can be of importance for Siglec binding. At least, we hypothesize that the protein backbone can provide specific binding context for Siglecs (**Figure 2G**) and multivalency for high avidity binding can be created by dense sialoglycan presentation on a protein backbone or *via* dense glycan clusters at the cell membrane, which we will touch upon later in more detail.

### Regulation of Siglec Ligands

A remaining knowledge gap is how expression of specific Siglec ligands is regulated within the TME. Multiple factors might play a role here. For instance, the hypoxic environment could influence glycan composition. Human cancer-associated gangliosides have been observed to incorporate more Neu5Gc into their glycans than non-transformed cells (105). Gangliosides are a special class of glycosphingolipids – glycans attached to a ceramide anchor - that contain at least one sialic acid residue (106). Due to a deletion in the *Cmah* gene, humans are incapable of synthesizing Neu5Gc and are dependent on dietary Neu5Gc (107–109). The increase in ganglioside associated Neu5Gc was suggested to be associated with the hypoxic environment of tumors. Potentially, Siglec binding or loss of binding due to Neu5Gc incorporation can alter signaling in the TME.

Interestingly, hypoxia was shown to induce transcription of the sialic acid transporter Sialin (SLC17A5), which mediates transport of external sialic acid into the cell (110). Moreover, hypoxia was reported to influence expression of sialyltransferases (111, 112). Therefore, Siglec binding could be influenced by the hypoxic environment by adjustment of the main type of sialic acid that is incorporated in sialoglycans, but future studies should elucidate how this exactly affects Siglec activation. Besides gene expression, also enzyme activity of for example specific sialyltransferases could influence incorporation of the type of sialic acid, although additional research is required to further study enzyme activity within the TME and its influence on Siglec activation.

The shift in metabolism that is accompanied with malignant transformation adds an additional layer to regulation of glycosylation in the TME (113). HIF-1α is a key player in regulating cellular energetics within the hypoxic TME as it causes enhanced glucose uptake, which is not only required to generate ATP *via* various biosynthetic pathways, but it is also an important glycosylation precursor (55, 114, 115). HIF-1α suppresses the tricarboxylic acid cycle, ultimately preventing generation of UDP-N-Acetylglucosamine (UDP-GlcNAc), which is required for N-glycan branching (116, 117). Besides, hypoxia also influences sialylation, as it activates biosynthesis of CMP-NeuAc nucleotides through conversion of UDP-GlcNAc to ManNAc by GNE (118). This eventually results in enhanced overall cell surface sialylation. Overall, the changed metabolism that is observed in the TME is intertwined with aberrant glycosylation patterns, and future research should elucidate how this specifically affects Siglec signaling within this hypoxic TME.

**Figure 3:**
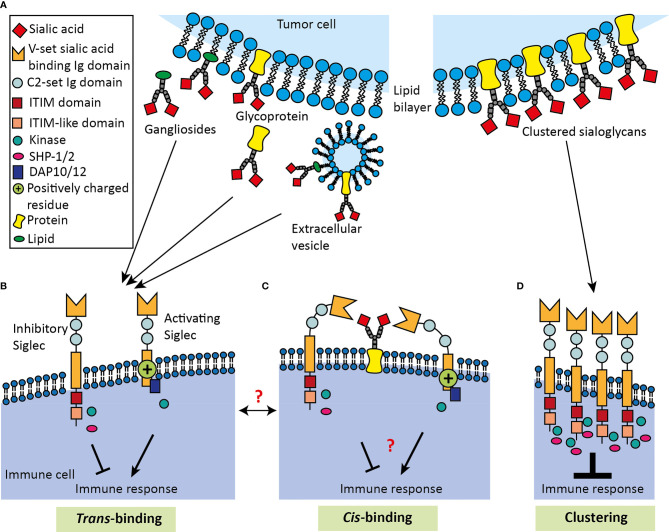
Schematic overview of Siglec binding to *cis*- and *trans*-ligands. **(A)** Tumor derived Siglec *trans*-ligands comprise glycolipids and glycoproteins, which can either be membrane-bound, secreted or present on EVs. Inhibitory and activating Siglecs can bind ligands either in **(B)** *trans*-fashion or in **(C)** *cis*-fashion. Siglecs bind their ligand using the V-set sialic acid-binding Ig domain, which is positioned away from the membrane by a variable number of C2-set Ig domains. Siglecs can bear ITIM and ITIM-like domains, which lead to inhibition of the immune response. In contrast, activating Siglecs can carry a positively charged residue in the transmembrane domain, which can associate with DAP10/12 activating transmembrane adaptor proteins to activate the immune response. *Cis*-ligand interactions probably contribute to immune homeostasis, but remain largely unknown and the dynamics of Siglecs binding in *trans* or *cis* mode requires more investigation. **(D)** Multivalent ligands are capable of clustering Siglecs to induce signaling, causing an intracellular accumulation of signaling molecules that eventually results in enhanced immune modulation.

Tumor cells have been demonstrated to release EVs able to modulate immune cell activation in the TME (119). Recently, the presence of Siglec ligands on EVs has been shown, which could be an additional manner in which tumor cells influence Siglec ligand expression within the TME (**Figure 3A**) (120). For example, Dusoswa et al. showed that isolated EVs (including exosomes and small membrane budded vesicles) of glioblastoma cell lines specifically express ligands for Siglec-9 (120). Moreover, exosomes derived from ovarian ascites fluid from cancer patients express GD3 on their surface (121). Shenoy et al. reported that expression of GD3 on liposomes inhibits T cell activation in a sialic acid-dependent way. GD3 has been reported to be a ligand of Siglec-7, which is expressed on subsets of T cells, and might be activated by GD3 positive exosomes (122–125). These data imply that T cell inhibition by GD3 can at least in part be Siglec-mediated, but this finding needs further experimental confirmation.

Another interaction with exosomes has been reported for Siglec-1 that is expressed on macrophages and dendritic cells. Siglec-1 is an atypical Siglec, as it has no intracellular signaling motif and has 16 C2 Ig domains that extend the V-set domain far away from the cell membrane (1). Although Siglec-1 does not have intracellular motifs and is mostly described in uptake of α2-3-sialylated proteins or particles, multiple studies have shown that Siglec-1^+^ macrophages exert immunosuppressive effects (43, 46, 126, 127). Functionally, Siglec-1 expression on macrophages has been described to be important for antigen transfer to dendritic cells or T cells (43, 46, 127). An *in vivo* mouse study demonstrated that Siglec-1^+^ macrophages in tumor-draining lymph nodes bind tumor-derived EVs to physically block further dissemination and subsequent lymphocyte activation (128). Black et al. reported that Siglec-1 also binds a special type of EVs, apoptotic bodies, derived from the EL4 lymphoma cell line (129). Importantly, they showed *in vivo* that *siglec*-1^-/-^ mice had an increased cytotoxic T cell response to apoptotic vesicles displaying ovalbumin protein than wild type C57BL/6. Thus, the data show that Siglec-1 activation could be enhanced by tumor cells secreting EVs displaying Siglec-1 ligands. However, it remains unknown whether Siglec-1 ligands are actively incorporated into membranes of EVs, or whether this is a passive process. Moreover, many factors regarding Siglec signaling induced by tumor-derived EVs remain unknown, such as the strength and kinetics of the activation and how these factors compare to Siglec activation induced by ligands on tumor cells. Furthermore, systemic transport of tumor-derived EVs and the resulting effect of these on Siglec activation outside the TME also requires further research, as well as the comparison between EVs derived from tumors and from healthy tissue.

In summary, Siglec ligand expression within the TME can be affected by various factors, such as hypoxia, its associated change in metabolism and the secretion of Siglec ligand-containing EVs. However, additional research is required to elucidate the contribution of these factors and possible others that have yet to be determined.

## Siglec Clustering and Multivalent Ligands

Immune inhibitory Siglec signaling requires a few steps that are similar to induction of signaling by PD-1. First of all, PD-1 receptors have been described to require clustering to initiate signaling and literature now supports the idea that Siglecs require clustering as well (**Figure 3D**) (3, 130). Inhibitory Siglecs harbor ITIM domains like the PD-1 receptors to which kinases get recruited upon clustering and activation to phosphorylate the ITIM domains (1, 131). Research has previously shown that intracellular kinases can locally accumulate at receptor clusters, which might also be the case for Siglec receptors (132). Lastly, SHP-1/2 phosphatases are recruited intracellularly to the clusters and these will cause dephosphorylation of downstream molecules to inhibit immune cell activation (133). Localization of the clusters of PD-1 receptors to T cell receptors (TCRs) enhances efficiency of PD-1 mediated suppression, and this might also be true for Siglecs and their downstream targets (130). Here, we will discuss the current evidence for Siglec clustering and that multivalent ligands can be bound by clusters of Siglec receptors.

### Siglec Clustering

It has been observed that Siglecs can form foci (such as Siglec-E on neutrophils) or accumulate at cell-cell contact sites, like the Siglec-9 accumulation on neutrophils that was observed at contact sites with LS180 and A549 carcinoma cells (60, 134). Furthermore, several studies used antibodies to cross-link Siglecs on the cell membrane to induce a functional effect (135–138). A typical example that demonstrates the relevance of Siglec clustering is given by Siglec-2. Siglec-2 binds *cis*-ligands resulting in cluster formation that inhibits B cell receptor signaling, which we will discuss in more detail in the section Siglec binding to *cis-*ligands (139–142). Another example was given by Ikehara et al., who transfected Jurkat T cells with Siglec-7 or -9 and observed clustering and partial co-localization of these receptors with CD3 of the TCR (125). Subsequently, they found that Siglec expression leads to reduced phosphorylation of molecules downstream of the TCR following TCR engagement.

### Multivalent Siglec Ligands

The monovalent binding affinity of a Siglec for a sialoglycan ligand is relatively low (K_d_ of 100-300 μM) (4, 8, 59). Multivalent interactions, the simultaneous binding between clustered receptors and multimeric ligands, increases the binding affinity between Siglecs and sialoglycans as has been shown for Siglec-2 clusters (143–149). For instance, lipid rafts that contain many gangliosides can be seen as a multivalent Siglec ligand. Accordingly, Nicoll et al. reported clustering of unmasked Siglec-7 on NK cells at the contact site with ganglioside GD3-synthase transfected P815 cells (150).

Multivalent Siglec ligands can be formed at the cell surface through clustering of individual sialoglycoproteins or lipids or are formed by densely glycosylated protein such as the mucins (151–155). Mucins are a family of large secreted and membrane-bound glycoproteins consisting for up to 60% of tandem repeat domains formed by repeating serine, threonine and proline sequences (156, 157). Both serine and threonine are acceptors of O-GalNAc-type glycosylation resulting in dense glycosylation of mucins that can account for up to 80% of their total mass. Gel-forming mucins such as MUC2 and MUC5AC form a barrier at the epithelial surfaces and house the microbiome, and membrane-bound mucins have many functions in cell signaling and cellular interactions (157).

CD43 is another example of multivalent Siglec ligand (158, 159). Wisnovsky et al. discovered CD43 as a multivalent ligand for Siglec-7 on the K562 chronic myeloid leukemia cell line using a genome-wide CRISPR screen (158). CD43, or sialophorin, is a mucin-type protein harboring a heavily O-glycosylated extracellular domain (160, 161). This study showed that CD43 can relocalize Siglec-7 on NK cells to the immunological synapse with the K562 cells. In parallel, Yoshimura et al. identified CD43 on K562 cells as Siglec-7 binding partner using biochemical techniques (159).

In summary, binding of Siglecs to their ligands can be strengthened by multivalent interactions and in line with this, several multivalent ligands for Siglecs have been identified.

### Multivalent Siglec Ligands Present in the TME

Mucins are overexpressed and aberrantly glycosylated in many types of cancer (162, 163). Several studies have reported Siglec binding to (cancer-associated) mucins suggesting that they can mediate communication between the epithelial surface and the immune system and that they could alter the anti-tumor immune response (151–154). For instance, MUC1 that is overexpressed in many cancer types has been reported to interact with Siglec-1 and Siglec-4, mediating adhesion between Siglec-4 expressing Schwann cells and MUC1 expressing pancreatic cells (152, 154). Beatson et al. reported that the cancer-associated sialyl T glycoform of MUC1 binds with Siglec-9 on myeloid cells and that this interaction can induce a tumor-associated macrophage phenotype (151, 164). Sialyl T-MUC1 instructed macrophages secrete soluble factors (IL-6, M-CSF, PAI-1) associated with tumor progression and display enhanced levels of the inhibitory receptor PD-L1 (151, 164). Moreover, these mucins enabled tumor-associated macrophages to inhibit T cell proliferation. Recently, the interaction of the human Siglecs with a large panel of recombinant mucins and mucin-like proteins decorated with defined O-GalNAc-type glycans was dissected (9). Although Siglec-9 interactions with sialyl T-MUC1 were not confirmed, this study revealed Siglec-4, -7, and -15 interactions with mucins that were largely determined by the mucin O-glycan pattern and type.

Another multivalent Siglec ligand that has been found on tumor cells or secreted in the TME is LGALS3BP (Mac-2 binding protein) (165, 166). LGALS3BP is a heavily N-glycosylated protein and Siglec-5, -9, and -10 have been shown to bind it (166). Importantly, recombinant LGALS3BP was demonstrated to inhibit neutrophil activation and siRNA-mediated reduction of LGALS3BP expression in HT-29 colon cancer cells increased neutrophil-induced apoptosis of the HT-29 cells (166). Interestingly, Koths et al. have shown before that LGALS3BP can form multimeric complexes (167). These data suggest a role for complex formation of Siglec ligands to offer a multivalent Siglec ligand to activate Siglec receptors.

Altogether, Siglecs can accumulate at the cell surface to induce a functional effect. Multivalent ligands increase binding affinity between Siglecs and sialoglycans. Classic example of tumor associated multivalent Siglec ligands are mucins. Smaller Siglec ligands, such as CD24 for Siglec-10, however, have also been identified in the TME that are less likely to act as multivalent ligand (106–108). Potentially, clustering of such ligands within the cell membrane could result in local presentation of Siglec ligands in a multivalent manner. In the same way, glycolipids could possibly organize in lipid rafts to activate Siglecs on the same cell (*cis*) as well as on opposing cells (*trans*) (59).

It would be interesting to study whether there is a critical amount of specific interactions and/or Siglec clustering that is required to trigger Siglec signaling. Also, comparison of the strength and kinetics of Siglec activation by ligands that either offer a multivalent or monovalent ligand would contribute to the current knowledge on Siglec activation by multivalent ligands and would aid in the rational development of Siglec targeting therapeutics. We hypothesize that the multivalency of Siglec ligands can have multiple functions, for example to induce Siglec clustering and thereby have a functional effect or to outcompete *cis*-ligands with a lower affinity. Nevertheless, more research is required to study these hypotheses and elucidate whether and how monovalent ligands can activate Siglecs.

## The Function of Siglec Binding to *Cis*-Ligands

In order for Siglecs to interact with *trans*-ligands (**Figure 3B**), for instance on tumor cells, Siglecs have to be available for binding. Cells expressing Siglecs also express themselves sialoglycan ligands on their membrane that can interact in *cis* thereby ‘masking’ the V-set binding site (59) (**Figure 3C**). This has been shown for most Siglecs and potentially lowers the threshold for Siglec signaling by *trans*-interactions (45, 150, 168–172). Naturally, masking interactions are overcome by the dynamic on/off binding of Siglecs with their ligands, in particular for lower (monovalent) affinity ligands, that enables binding to higher affinity ligands (1, 4). Noteworthy, masking can also be abolished by sialidases or experimentally by treatment with sialylation inhibitors (17, 173). Furthermore, the C2-set domain repeats allows orientation of the V-set domain away from *cis-*ligands which likely contributes to recognition of *trans*-ligands. The 16 C2 Ig domains that orient the V-set domain of Siglec-1 into the extracellular space were suggested to enable Siglec-1 to mediate intercellular interactions. Still, interactions of Siglec-1 with cis-ligands has been observed (168, 174). However, it remains unknown whether *cis*-ligands only mask Siglec-1 or also activate the receptor. Variation in the C2-set repeat numbers between the Siglecs possibly determines the sensitivity of individual family members for the *cis-* and *trans*-ligands and also endogenous membrane and secreted sialidases (e.g. NEU1, NEU3).

Glycosylation changes in the cell have also been suggested to regulate Siglec binding dynamics for *cis-* and *trans*-ligands (169, 175). Masking/unmasking dynamics under physiological conditions and particularly in the TME and consequences for signaling and immune regulation is likely of key importance in Siglec biology.

### Effect of Siglecs Binding to *Cis*-Ligands

One of the main knowledge gaps regarding *cis-*ligands is whether Siglecs can only be activated by *trans-* ligands or also by *cis-*ligands. The best studied Siglec receptor in terms of *cis*-interactions is Siglec-2, which is a receptor intracellularly harboring multiple ITIMs, an ITIM-like domain and a Grb2 binding motif (1, 176). Siglec-2 and B cell receptor (BCR) are present on the membrane as clusters that partially overlap (139). Co-clustering was found to be highly important for Siglec-2-mediated inhibition of BCR signaling as increased clustering of Siglec-2 with the BCR inhibits BCR signaling (139, 177). Siglec-2 is scavenged away from the BCR by *cis*-ligands on neighboring sialylated Siglec-2 receptors present on the same cell membrane leading to enhanced BCR activity (139–142). Collins et al. demonstrated that Siglec-2 on B cells can redistribute to the sites of cell contact with other lymphocytes, despite the presence of *cis*-ligands (178). This indicates that clustered patterns of Siglecs have to be tightly regulated and are affected by both *cis*- and *trans*-ligands (140, 141).

Human Siglec-2 has a high affinity for Neu5Ac as sialic acid and a 6-O-sulfate on the GlcNAc in the underlying glycan (175, 179). When human B cells are activated, the 6-O-sulfotransferases needed for the Siglec-2 ligands are downregulated. This results in a decrease in interactions between Siglec-2 and *cis*-ligands and therefore Siglec-2 becomes available to inhibit the BCR. Accordingly, *in vivo* mouse studies established that mice that lack the α2-6 sialyltransferase *St6gal1* and mice with a mutation of the critical Arginine in the V-set domain of Siglec-2 both showed enhanced BCR inhibition. Murine Siglec-2 on B cells has a similar mechanism to inhibit BCR signaling. Murine Siglec-2 has a preference for Neu5Gc, rather than Neu5Ac (180). It is unmasked by downregulation of the hydroxylase that is responsible for the conversion of Neu5Ac to Neu5Gc, eventually resulting in a reduction of Siglec-2-*cis*-interactions and enhanced BCR inhibition (175, 181). Nevertheless, despite the ability of *cis*-ligands to prevent Siglec-2 to inhibit BCR signaling, Siglec-2 is always able to bind *trans*-ligands on an opposing cell (178, 182, 183). This interaction causes Siglec-2 to translocate to the immunological synapse, where it can inhibit BCR signaling (184, 185).

A recent study by Ballet et al. (2021) has further established Siglec-2 signaling on B cells induced by *cis*-ligands (186). Here, Siglec-2 was shown to associate with β_7_ integrin in a sialic acid-dependent manner. Next, recruitment of SHP-1 to Siglec-2 was demonstrated to inhibit β_7_ integrin endocytosis and restrain β_7_ integrin phosphorylation at the cell surface. The β_7_ integrin complexes with the α_4_ integrin, and this complex is involved in homing B lymphocytes to gut-associated lymphoid tissue, where B cells are being activated. Indeed, the Siglec-2-SHP-1 axis was shown to enhance B cell homing to the gut-associated lymphoid tissue.

Another study on Siglec signaling induced by *cis*-ligands has recently been performed by Delaveris et al. (2021) (187). Applying synthetic lipid-tethered glycopolypeptides that inserted into cell membranes in combination with Förster resonance energy transfer analysis revealed binding between Siglec-9 and glycopolypeptides in *cis*. Strikingly, Siglec-9 binding to ligands in *cis* reduced LPS-mediated MAPK signaling, cytokine secretion and phagocytosis in macrophages, while the same ligand used in *trans* did not. Of note, these studies on Siglec interactions with *cis*-ligands have not been performed within the context of the TME. Activation of Siglecs by *cis*-ligands in the TME therefore remains to be elucidated.

### Relevance of *Cis*-Ligands in the TME

Although it has been established that Siglecs are masked by *cis*-interactions, for many Siglecs the biological role of these interactions is poorly understood. Varki and Angata (2006) proposed that *cis-*interactions set a threshold for recognition of *trans*-ligands with higher affinity or to monitor sialylation on the own cell surface (188). C*is*-interactions could have a gatekeeper function blocking insubstantial interactions and signaling enabling sensing of high affinity or avidity Siglec ligands that lead to a biologically relevant signaling outcome. C*is*-interactions, on the other hand, may exert baseline signaling which in case of the inhibitory Siglec members may support a resting state or return to a resting state after activation. Understanding the physiological relevance of Siglec *cis-*interactions and possible signaling requires further investigation. Likewise, the relevance of Siglec *cis*-interactions in the TME is largely unknown.

It is conceivable that tumor-associated *trans*-ligands outcompete *cis*-ligands either due to higher affinity or avidity for instance. The malignant transformation could potentially guide expression of specific glycan/glycoprotein or higher expression levels and clustering of *trans-*ligands. Altered biosynthesis, or degradation of *cis-*ligands on Siglec-expressing immune cells upon infiltration into the TME could further increase Siglec activation on immune cells upon encounter with tumor cells. For instance, it has been reported that T cell activation can result in a loss of sialylation on core 1-O glycans (189–191). Similarly, IL-2 stimulation of NK the cell line NK-92 reduced gene expression of the sialyltransferases ST8SIA1, ST6GAL1, and ST3GAL1, but increased overall α2-6-linked sialic acids and poly sialic acid likely due to an increase in the expression of poly sialic acid carrier molecule NCAM/CD56 (192). Occurrence of such alterations in glycosyltransferase and sialyltransferase expression in the TME, but also sialidase expression or changes in nucleotide sugar metabolism could thus alter *cis*-ligand expression. Together with the observed changes in immune cell phenotype like the enhanced Siglec expression on T cells and macrophages in response to factors in the TME, this would enable strong *trans*-interactions with sialoglycans on tumor cells and modulate the immune cell function (11, 28, 94). Clearly, multiple aspects need further investigation to understand how Siglec *cis*-interactions in the TME are altered and the biological effects thereof.

### Challenges for the Study of *Trans*- and *Cis*- Siglec Interaction Dynamics

The dynamics of Siglec binding to *trans-* and *cis-*ligands is emerging as a key event in Siglec biology determining their clustering, signaling and biological effects. Studying these dynamics, however, is challenging. To study *cis-*binding-mediated Siglec activation, studies have installed lipid-conjugated glycopolypeptides into cell membranes (187, 193, 194). Similarly, to study *trans-*binding-mediated Siglec activation, Siglec-ligand containing liposomes have been used (195–197). However, addition of Siglec ligands in such manners requires careful interpretation, as this can lead to overexpression of unnatural sialic acids that can be recognized by Siglecs either in *cis* or *trans* and does not represent a natural situation. Besides, novel tools have been developed to remove sialic acids from the cell membrane to assess Siglec binding in either *cis* or *trans* manner. Sialic acid removal from the cell membrane can be established using sialidase or sialic acid mimetics that can enter the cell to inhibit sialyltransferases and thereby prevent transfer of sialic acid onto glycan chains (4, 173).

Nevertheless, altering cellular sialylation by different means can co-affect other factors. For instance, a recent study by Edgar et al. has provided an example that abrogation of sialic acid expression can result in Siglec-independent effects (198). They have shown that the costimulatory molecule CD28 on T cells can bind to sialoglycans in *cis* and in *trans*. The interactions of CD28 with *cis*-ligands limited binding of CD28 to its *trans* protein ligands CD80 and CD86 on antigen-presenting cells thereby negatively affecting co-stimulation. This example illustrates that also the function of non-Siglec receptors can be affected by abrogation of sialic acid expression. Moreover, removal of sialic acids will expose galactose, which can be recognized by galectins, which can modulate the immune response and affect tumor development (199). Similar mechanisms may be in place for other sialoglycan binding receptors, such as factor H and selectins (6). Furthermore, removal of the negatively charged sialic acid molecules could affect total charge of the cell and might therefore affect cellular interactions. Indeed, it was demonstrated that diminished sialic acid expression caused enhanced tumor cell killing by cytotoxic CD8^+^ T cells, which was explained at least in part by increased clustering of the T cells with tumor cells (200).

Additionally, studies have genetically manipulated the glycosylation machinery to modify sialylation, for example *via* genetic glycoengineering with inducible sialyltransferase expression to tune Siglec ligand expression (11, 201–203). However, this might also affect other processes, as Kohnz et al. have found that knockdown of cytidine monophosphate N-acetylneuraminic acid synthase, an enzyme required for sialic acid activation to be incorporated in glycans, affected levels of more than 200 other gene transcripts including oncogenes (204).

Overall, results have to be carefully interpreted when interrupting Siglec ligands, as these type of experiments are likely to have various side effects.

## Concluding Remarks

The Siglec-sialoglycan axis emerges as important regulator of immune cell-tumor cell interactions in the TME that co-determines the outcome of tumor immunity (17). Qualitative and quantitative changes in Siglec ligand expression as well as Siglec receptors on immune cells in the TME have been linked to immune evasion. However, unraveling the biological effects and consequences of Siglec signaling in the TME remains challenging for several reasons. First, healthy cells and cancer cells can produce a highly diverse Siglec interactome that is formed by the sialoglycan structures, their modifications, their display on particular glycoproteins and glycolipids as well as multivalent higher order binding patterns created by protein glycosylation density and ligand clustering. Deducing the specific and biological meaningful *cis-* and *trans*-interactions of each of the individual Siglec family member is currently ongoing aided by recent technological advances with recombinant (multimeric) Siglec probes, proximity labeling approaches, cell-based glycan arrays, and genome-wide CRISPR screens (9, 22, 158, 173, 205).

Second, the expression patterns and membrane organization of most Siglecs on immune cells in the periphery and the TME (and other tissues) are not well understood. Siglec-2 is the best studied Siglec in that context and shows the importance of *cis-*interactions in tuning B cell receptor activation. Siglec-2 interactions with high avidity ligands in *trans* and downregulation of *cis-*ligands result in clustering around the B cell receptor and potent inhibitory signaling sufficient to block B cell activation (139–142). The membrane organization and interaction partners, recruitment to the membrane, internalization and recycling kinetics of the other Siglecs are less well understood, but should move more into focus as they likely determine their individual signaling modes.

Third, still relatively little is known regarding the signaling of Siglecs and the downstream molecular targets and effects. The immune inhibitory Siglecs that recruit SHP-1 or SHP-2 after ITIM phosphorylation are best described, but the molecular and cellular consequences of their signaling are not fully understood. Likewise, signaling of the activating Siglecs-14, -15, and -16 *via* adaptor proteins needs further dissection as well as other potential signaling residues (Grb2, Fyn kinase sites). Next to understanding the consequences of Siglec signaling and their integration with other immune signaling pathways, systems to determine whether ligand binding equals signaling are required. Studies with Siglec-Fc chimeras inform on binding, but this does not necessarily correlate with signaling (22). Suematsu et al. developed a reporter system for direct measurement of Siglec activation using a receptor consisting of the extracellular Siglec domains and the transmembrane and intracellular domains of the CD3ζ chain (206). Such cell-based assays could allow direct measurement of Siglec activation in response to a ligand, measure effects of signaling kinetics with different (multivalent) ligands, and shed light into signaling through *trans-* and *cis*-ligand binding dynamics.

Finally, further insight into general aspects of Siglec biology is needed to advance our currently limited understanding of inhibiting and activating Siglec signaling in the TME and the role that hypoxia, altered metabolism and cytokines play in regulating *cis*/*trans*-Siglec ligand and Siglec expression that guide cell-cell communication in the TME. The identification of Siglecs as important immune checkpoints in the TME implies that blocking monoclonal antibodies or other strategies to abolish Siglec-sialic acid interactions and signaling can boost anti-tumor immunity (16, 17, 28, 94). Further research into the diverse Siglec signaling modes in the TME may turn out to be highly awarding to uncover the impact of therapeutic Siglec targeting on the efficacy of cancer immunotherapy.

## Author Contributions

EH designed and wrote the review, CB edited the review and LC and GA designed, supervised and edited the review. All authors contributed to and approved the submitted version.

## Funding

This work was supported by a Radboudumc grant awarded to EH and grants from the Dutch Cancer Foundation awarded to GA, K.C.M. Santegoets and P. Wesseling (KWF 11266) and GA, LC and DG (KWF 2021-1 13373).

## Conflict of Interest

The authors declare that the research was conducted in the absence of any commercial or financial relationships that could be construed as a potential conflict of interest.

## Publisher’s Note

All claims expressed in this article are solely those of the authors and do not necessarily represent those of their affiliated organizations, or those of the publisher, the editors and the reviewers. Any product that may be evaluated in this article, or claim that may be made by its manufacturer, is not guaranteed or endorsed by the publisher.
